# Randomized Trial of Pegmolesatide for the Treatment of Anemia in Patients With Nondialysis CKD

**DOI:** 10.1016/j.ekir.2024.12.002

**Published:** 2024-12-06

**Authors:** Jianteng Xie, Aicheng Yang, Hongyu Qiu, Xiaomei Peng, Wanhong Lu, Xiangyang Huang, Qinkai Chen, Aimin Zhong, Shuifu Tang, Qin Wang, Chuan Li, Liangliang He, Xiaohong Jia, Anran Ma, Fan Wang, Xueqing Yu

**Affiliations:** 1 Department of Nephrology, Guangdong Provincial People’s Hospital (Guangdong Academy of Medical Sciences), Southern Medical University, Guangzhou, China; 2 Department of Nephrology, Wuyi Traditional Chinese Medicine Hospital, Jiangmen, China; 3 Department of Nephrology, West China Medical Center of Sichuan Medical University, Chengdu, China; 4 Department of Nephrology, The People's Hospital of Guangxi Zhuang Autonomous Region, Nanning, China; 5 Department of Nephrology, The First Affiliated Hospital of Xi’an Jiao Tong University, Xi’an, China; 6 Department of Nephrology, Liuzhou Workers' Hospital, Liuzhou, China; 7 Department of Nephrology, The First Affiliated Hospital of Nanchang University, Nanchang, China; 8 Department of Nephrology, Jiangxi Provincial People's Hospital, Nanchang, China; 9 Department of Nephrology, The First Affiliated Hospital, Guangzhou University of Traditional Chinese Medicine, Guangzhou, China; 10 Department of Nephrology and Rheumatology, Fengxian District Central Hospital, Shanghai, China; 11 Hansoh Pharmaceutical Group Co, Ltd, Shanghai, China

**Keywords:** anemia, chronic kidney disease, epoetin alfa, nondialysis, pegmolesatide

## Abstract

**Introduction:**

Pegmolesatide has been recently approved for treating anemia in chronic kidney disease (CKD) patients in China. We presented the results of the pivotal study conducted in patients with nondialysis-dependent (NDD)-CKD with anemia.

**Methods:**

This randomized, active-controlled, open-label, noninferiority phase 3 study was conducted across 38 centers in China. Eligible patients were randomly assigned to receive subcutaneous injection of pegmolesatide in the upper arm once every 4 weeks or epoetin alfa weekly or biweekly, with doses adjusted to maintain hemoglobin (Hb) level of 100 to 120 g/l. The primary outcome was the mean change in Hb level from the baseline during the efficacy evaluation period. Noninferiority of pegmolesatide to epoetin alfa was established if the lower limit of the 2-sided 95% confidence interval (CI) was ≥ −10 g/l.

**Results:**

A total of 173 patients received at least 1 dose of pegmolesatide (115 patients) or epoetin alfa (58 patients). During the efficacy evaluation period, the mean change in Hb from baseline level was 19.2 g/l in the pegmolesatide group and 15.4 g/l in the epoetin alfa group with a between-group difference of 3.8 g/l (95% CI: 0.7–6.9). The incidence of adverse events (AEs) and serious AEs (SAEs) were similar between groups, with hypertension being the most reported AE related to the study drug. No drug-related hypersensitivity reactions or fatal events were observed.

**Conclusion:**

Pegmolesatide demonstrated comparable efficacy to epoetin alfa in elevating and maintaining Hb levels in patients with NDD-CKD with anemia without new safety concerns (ClinicalTrials.gov identifier: NCT03903809).

## Graphical abstract

**Figure:**
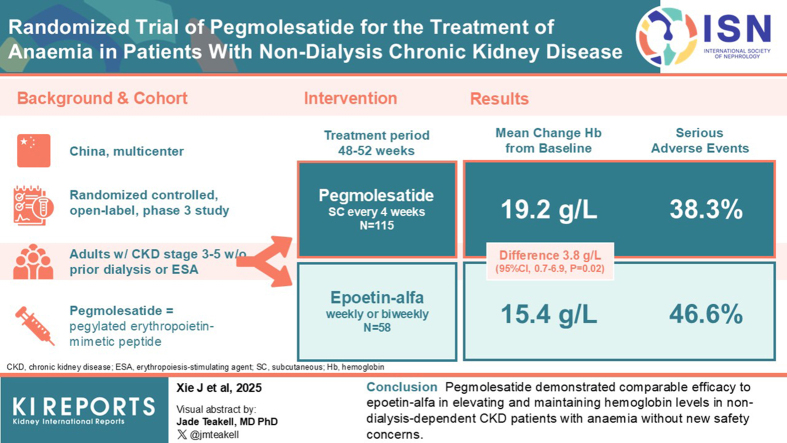

Renal anemia is a frequent complication caused by reduced erythropoiesis in CKD. In China, the prevalence of anemia is > 50% in CKD,^1^ and even exceed 90% in stage 5 CKD.^2^ Erythropoiesis-stimulating agents (ESAs) such as darbepoetin alfa and continuous erythropoietin receptor activator are conventional treatment of renal anaemia^3^; however, high-dose ESAs may have safety concerns about the increased risk of cardiovascular events.

Recently, hypoxia-inducible factor–prolyl hydroxylase inhibitors have been approved as a new class of oral alternatives to conventional ESAs for treating anemia in China. However, the involvement of hypoxia-inducible factor in various pathway regulations may also potentially lead to related AEs, such as tumor development,^4^ exacerbation of polycystic kidney disease,^5^^,^^6^ renal fibrosis,^7^, ^8^, ^9^ hyperkalemia,^10^^,^^11^ and pulmonary hypertension.^12^ Therefore, a new drug with improved treatment compliance and better safety profile that offers a higher response rate is pressingly needed.

Pegmolesatide (HS-20039, previously known as pegolsihematide or EPO-018B) is a novel pegylated EPO-mimetic peptide developed by Hansoh Pharmaceutical Group Co, Ltd (Shanghai, China). Preliminary results from phase 2 trials have shown promising outcomes in maintaining target Hb levels in patients with CKD.^13^^,^^14^ Recently, based on the noninferiority of pegmolesatide to epoetin alfa in 2 pivotal phase 3 studies, one of which focused on the dialysis-dependent CKD population,^15^ pegmolesatide was approved by China National Medical Products Administration for the treatment of anemia in both patients with dialysis-dependent CKD and those with NDD-CKD. Here, we presented the results from another pivotal trial (NCT03903809) that evaluated the efficacy and safety of pegmolesatide in comparison with epoetin alfa, in Chinese NDD-CKD patients with anemia.

## Methods

### Study Design

**Figure 1:**
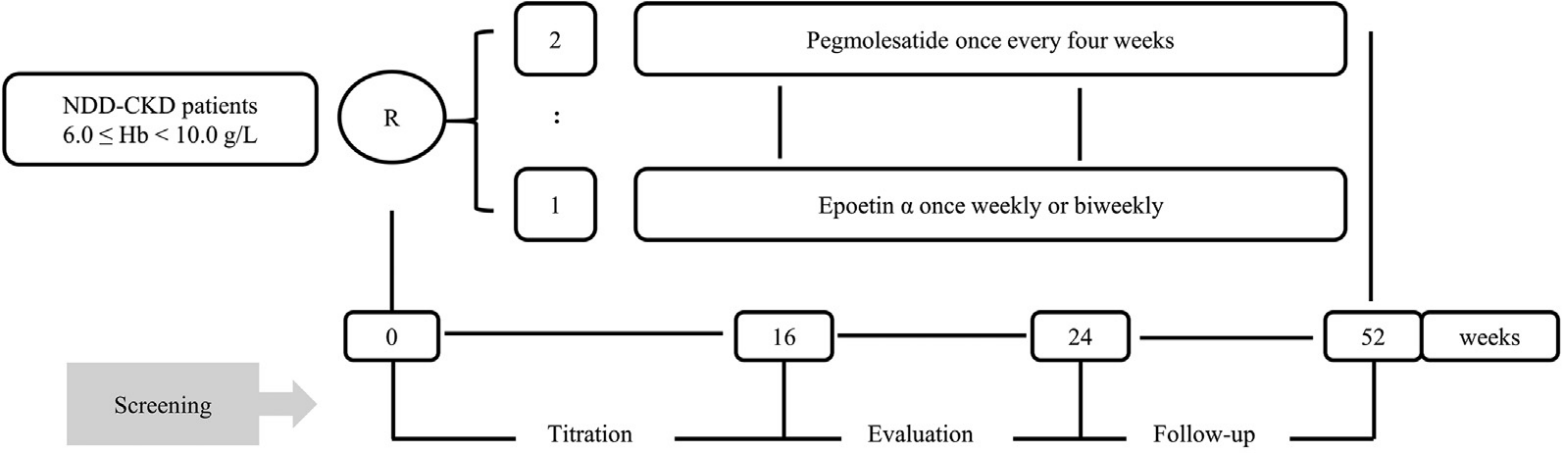
Study design. NDD-CKD, nondialysis-dependent chronic kidney disease.

This randomized, open-label, phase 3 noninferiority study aimed to assess the efficacy and safety of pegmolesatide compared with the active comparator, epoetin alfa, in patients with NDD-CKD with anemia, and was conducted from June 2019 to May 2022 across 38 centers in China (Supplementary Data S1). The trial consisted of 3 stages as follows: (i)a dose-titration period (weeks 0–16), (ii) an efficacy evaluation period (weeks 17–24), and (iii) an extended period (weeks 25–52) (Figure 1).

### Ethics Statement

The study protocol was approved by the local institutional ethics committee at each center and conducted in accordance with the Declaration of Helsinki and the International Conference on Harmonization Good Clinical Practice Guidelines. All patients provided their written informed consent to enroll in this study. This trial was registered with ClinicalTrials.gov under the identifier NCT03903809 (Trial name: Study of the Efficacy and Safety of Pegol-Sihematide for Anemia in Patients with NDD-CKD).

### Participants

All eligible patients enrolled in the study were aged between 18 and 70 years and had stage 3 to 5 CKD without previous dialysis or ESA treatment within 12 weeks before randomization. During screening, estimated glomerular filtration rate < 60 ml/min per 1.73 m^2^ and Hb value range of 60 ≤ Hb < 100 g/l were required. Patients were also required to have transferrin saturation ≥ 20% and serum ferritin levels ≥ 100 ng/ml, as well as normal levels of serum folate and vitamin B12 (Supplementary Data S2).

### Randomization and Masking

Participants were randomly assigned with a ratio of 2:1 to receive either pegmolesatide or epoetin alfa using an interactive web response system and a fixed-size block allocation with a block size of 6. The interactive web response system generated the randomization list and assigned patients to the next available treatment according to the randomization schedule. The interactive web response system allocated dispensing unit numbers for each patient, with the trial product dispensed by the site investigator or study coordinator at the trial site visits. Because this was an open-label study, no masking strategy was employed.

### Interventions

Eligible patients in the pegmolesatide group received an initial dose of 0.04 mg/kg, subcutaneously administered in their upper arm every 4 weeks until week 48, and those in the epoetin alfa group received an initial dose of 6000 IU per week, administered either weekly or biweekly, until the final dose before week 52. Throughout the trial, dose adjustment was performed every 4 weeks for 2 groups, to maintain the Hb levels within the target range of 100 g/l to 120 g/l. The maximum dose of the pegmolesatide administered by the subjects was 0.08 mg/kg, whereas the maximum dose of the epoetin alfa that patients received was 9000 IU/week.

### Outcomes

The primary efficacy end point was the mean change from the baseline in Hb level during the efficacy evaluation period. The secondary efficacy end points included the following: (i) the mean change from baseline in Hb level at each visit after baseline; (ii) the proportion of patients who maintained a mean Hb of 100 to 120 g/l during the efficacy evaluation period; (iii) the actual mean dose administrated for subjects with Hb levels maintained between 100 g/l and 120 g/l during the efficacy evaluation period; (iv) the time to achieve the first Hb response, defined as an increase of ≥ 10 g/l in Hb from baseline at any time point during the trial; and (v) the time to achieve the first Hb response target, which was defined as follows: for patients whose baseline Hb were ≥ 80 g/l, achieving an increase of ≥ 10 g/l in Hb from baseline and reaching an Hb level ≥ 100 g/l at any visit; for those with a baseline Hb < 80 g/l, achieving an increase of ≥ 20 g/l in Hb from baseline at any visit.

Safety assessments comprised of the number of AEs and SAEs, laboratory parameters, vital signs, electrocardiogram parameters, and pegmolesatide antidrug antibodies. A more comprehensive description of immunogenicity analysis is provided in the Supplementary Materials and Methods. An independent Clinical Endpoint Committee reviewed composite safety events (comprising all-cause death, stroke, and myocardial infarction) and other cardiovascular events (such as congestive heart failure requiring hospitalization and unstable angina requiring hospitalization).

### Statistical Analysis

A sample size of approximately 141 patients provides at least 90% power to show a between-group difference in the primary efficacy end point of −10 g/l and a common SD of 17 g/l at a 1-sided significance level of 0.025. Assuming a dropout rate of 15%, a total of 168 patients were planned to be enrolled in this study, with 112 cases receiving pegmolesatide and 56 receiving epoetin alfa.

The full analysis set (FAS) was used for demographic baseline analysis and efficacy analysis. It included all randomized patients who received at least 1 dose of treatment (analyses based on randomized treatment assignment). The safety set was used for safety analysis and included all patients who took at least 1 dose of treatment and had postdose safety records (analyses based on actual treatment assignment). The FAS and safety set were equivalent at data cut off for the analysis included in this study. All statistical analyses were performed using SAS (version 9.4 or higher).

The primary efficacy end point was evaluated using an analysis-of-covariance model, with baseline Hb level as a covariate. For missing data on the primary efficacy end point, imputation methods were used. If no Hb value was available during the efficacy evaluation period, the mean of the last 3 Hb tests during the dose-titration period was used. If neither the efficacy evaluation nor the dose-titration period had an Hb test, the baseline Hb level was used. The difference between treatment groups in least-squares means and its 2-sided 95% CI were calculated. Noninferiority was established if the lower limit of the 2-sided 95% CI was ≥ −10 g/l.^16^^,^^17^ Subgroup analyses of demographic and clinical characteristics were performed on the primary efficacy end point. Sensitivity analyses were also conducted. One sensitivity analysis used analysis-of-covariance model with a different imputation method (imputing missing mean Hb change from baseline as 0 g/l when no Hb value was available during the efficacy evaluation period). Another sensitivity analysis used mixed model repeated measures analysis of observed data without imputation of missing data. The fixed effects in the model included randomized treatment group, visit, treatment group by visit interaction, and baseline Hb level. An unstructured covariance matrix was assumed.

For the secondary efficacy end points, time to first Hb response and time to first Hb response target of 2 treatment groups were compared using log-rank test, and the hazard rates in the pegmolesatide group were compared with that in the epoetin alfa group using the Cox proportional hazards model with the baseline Hb value as covariate. The percentage of participants receiving pegmolesatide and epoetin alfa, maintaining their Hb level within the target range of 100 to 120 g/l during the efficacy evaluation period were compared using Cochran-Mantel-Haenszel test, including baseline Hb group (≤ 89 g/l, ≥ 90 g/l) as correction factor.

## Results

### Patient Characteristics

**Figure 2:**
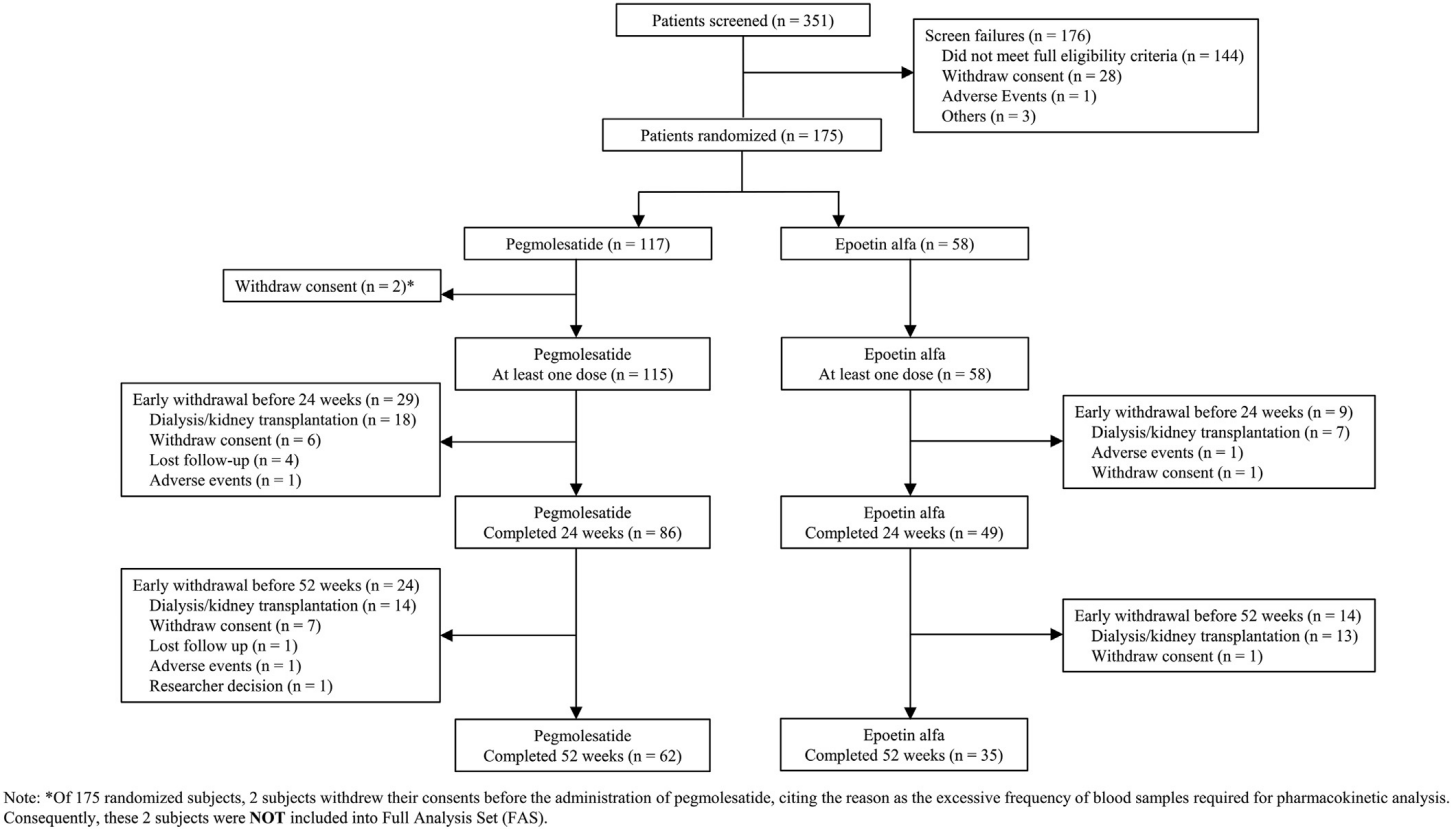
Flow diagram of patient disposition.

A total of 351 individuals from 38 research centers were enrolled in the study starting in June 2019. During screening, 176 cases (50.1%) were deemed ineligible. Among the 175 randomized participants, 173 individuals (115 in the pegmolesatide group and 58 in the epoetin alfa group) received at least 1 dose of the study drug and were included in both FAS and safety set. Among these, 135 participants (77.1% of the randomized individuals) completed the initial 24-week trial, with 86 in the pegmolesatide group and 49 in the epoetin alfa group. Eventually, 97 participants completed the full 52-week trial, with 62 patients in the pegmolesatide group and 35 patients in the epoetin alfa group (Figure 2).

Baseline demographic and disease characteristics of the study participants included in the FAS were balanced between treatment allocations (Table 1). The median CKD duration was 14.0 months in the pegmolesatide group and 13.4 months in the epoetin alfa group. Both groups had similar mean estimated glomerular filtration rate and distribution. The mean baseline Hb was 89.0 (SD: 10.0) g/l and 89.7 (SD: 10.8) g/l in the pegmolesatide and epoetin alfa groups, respectively.

**Table 1:** Baseline and demographic characteristics (FAS)

Characteristics	Pegmolesatide (*n* = 115)	Epoetin alfa (*n* = 58)
Age (yr), mean (SD)	51.7 (12.4)	53.6 (10.1)
Male, *n* (%)	48 (41.7)	29 (50.0)
Baseline Hb (g/l), mean (SD)	89.0 (10.0)	89.7 (10.8)
Baseline Hb groups, *n* (%)		
≤ 89 g/l	59 (51.3)	26 (44.8)
≥ 90 g/l	56 (48.7)	32 (55.2)
NYHA class, *n* (%)		
Class 0–I	77 (67.0)	42 (72.4)
Class II	36 (31.3)	16 (27.6)
Ferritin (ng/ml), median (IQR)	256.0 (159.4–446.6)	257.0 (154.2–461.0)
Transferrin saturation (%), median (IQR)	30.9 (24.9–38.2)	29.5 (25.6–40.4)
Baseline eGFR (ml/min per 1.73 m^2^), median (IQR)	16.4 (12.1–30.6)	17.1 (11.7–26.2)
Baseline eGFR groups, *n* (%)		
< 10 ml/min per 1.73 m^2^	18 (15.7)	8 (13.8)
≥ 10 and < 20 ml/min per 1.73 m^2^	58 (50.4)	30 (51.7)
≥ 20 ml/min per 1.73 m^2^	39 (33.9)	20 (34.5)
CKD course (mo), median (IQR)	14.0 (1.9–44.7)	13.4 (5.1–30.8)
History of cardiovascular risk factors, *n* (%)		
Hypertension	103 (89.6)	49 (84.5)
Diabetes	45 (39.1)	24 (41.4)
Hyperlipidemia	65 (56.5)	33 (56.9)
Coronary heart disease	18 (15.7)	7 (12.1)
Cerebrovascular disease	16 (13.9)	9 (15.5)
Heart Failure	4 (3.5)	4 (6.9)
Arrhythmia	10 (8.7)	3 (5.2)
Peripheral vascular disease	10 (8.7)	10 (17.2)
Thromboembolic disease	1 (0.9)	0
History of allergy, *n* (%)	1 (0.9)	0

CKD, chronic kidney disease; eGFR, estimated glomerular filtration rate; FAS, full analysis set; Hb, hemoglobin; IQR, interquartile range; NYHA, New York Heart Association.

Data are expressed in *n* (%), mean (SD), and median (IQR). eGFR was determined using CKD-Epidemiology Collaboration.

Based on the safety set, the median duration of drug exposure was 51.7 (interquartile range [IQR]: 23.9–52.0) weeks for the pegmolesatide group and 48.7 (IQR: 30.6–50.7) weeks for the epoetin alfa group. The actual median dose was 2.5 (IQR: 1.8–3.1) mg every 4 weeks and 4802.6 (IQR: 3571.8–7000.0) IU per week, respectively (Supplementary Table S1). Throughout the 52-week study period, the dosage of pegmolesatide remained stable within the range of 0.04 mg/kg (Supplementary Table S2), with fewer patients experiencing dosage adjustments (94/115, 81.7% vs. 56/58, 96.6%) and less frequent adjustments (mean [SD]: 2.4 [1.9] vs. 3.1 [1.7] times) compared with the epoetin alfa (Supplementary Table S3).

### Primary Efficacy End Point

In the FAS, the least squares mean changes from the baseline Hb level to the mean level during the efficacy evaluation period was 19.2 g/l for the pegmolesatide group and 15.4 g/l for the epoetin alfa group. The prespecified noninferiority criterion was met: the least squares mean difference between the groups was 3.8 g/l (95% CI: 0.7–6.9, *P* = 0.02). Additional sensitivity analyses and prespecified subgroup analyses on FAS were consistent with the primary efficacy results (Table 2 and Supplementary Figure S1).

**Table 2:** Mean change in Hb level from baseline to week 17 to 24 (g/l) (FAS)

Analysis	Pegmolesatide	Epoetin alfa	Difference	95% CI	*P* value
Primary analysis	19.2 (0.9)	15.4 (1.3)	3.8 (1.6)	0.7, 6.9	0.02
Sensitive analysis 1 (ANCOVA)	17.2 (1.0)	14.2 (1.5)	3.1 (1.8)	-0.5, 6.6	0.09
Sensitive analysis 2 (MMRM)	19.5 (1.1)	15.4 (1.5)	4.1 (1.8)	0.6, 7.7	0.02

ANCOVA, analysis of covariance; CI, confidence interval; FAS, full analysis set; Hb, hemoglobin; MMRM, mixed models for repeated measures.

Data are expressed with least-squares mean (SD). In the primary analysis, we used ANCOVA model with baseline Hb level as a covariate and imputed missing data as followed: If no Hb value was available during the efficacy evaluation period, the mean of the last 3 Hb tests during the dose-titration period was used. If neither the efficacy evaluation nor the dose-titration period had an Hb test, the baseline Hb level was used. In sensitivity analysis 1, we used the same ANCOVA model as the primary analysis, but with a different imputation method (imputing missing mean Hb change from baseline as 0 g/l when no Hb value was available during the efficacy evaluation period). In sensitivity analysis 2, we used MMRM without imputation of missing data. The fixed effects in the model included randomized treatment group, visit, treatment group by visit interaction, and baseline Hb level. An unstructured covariance matrix was assumed.

### Secondary Efficacy End Points

**Figure 3:**
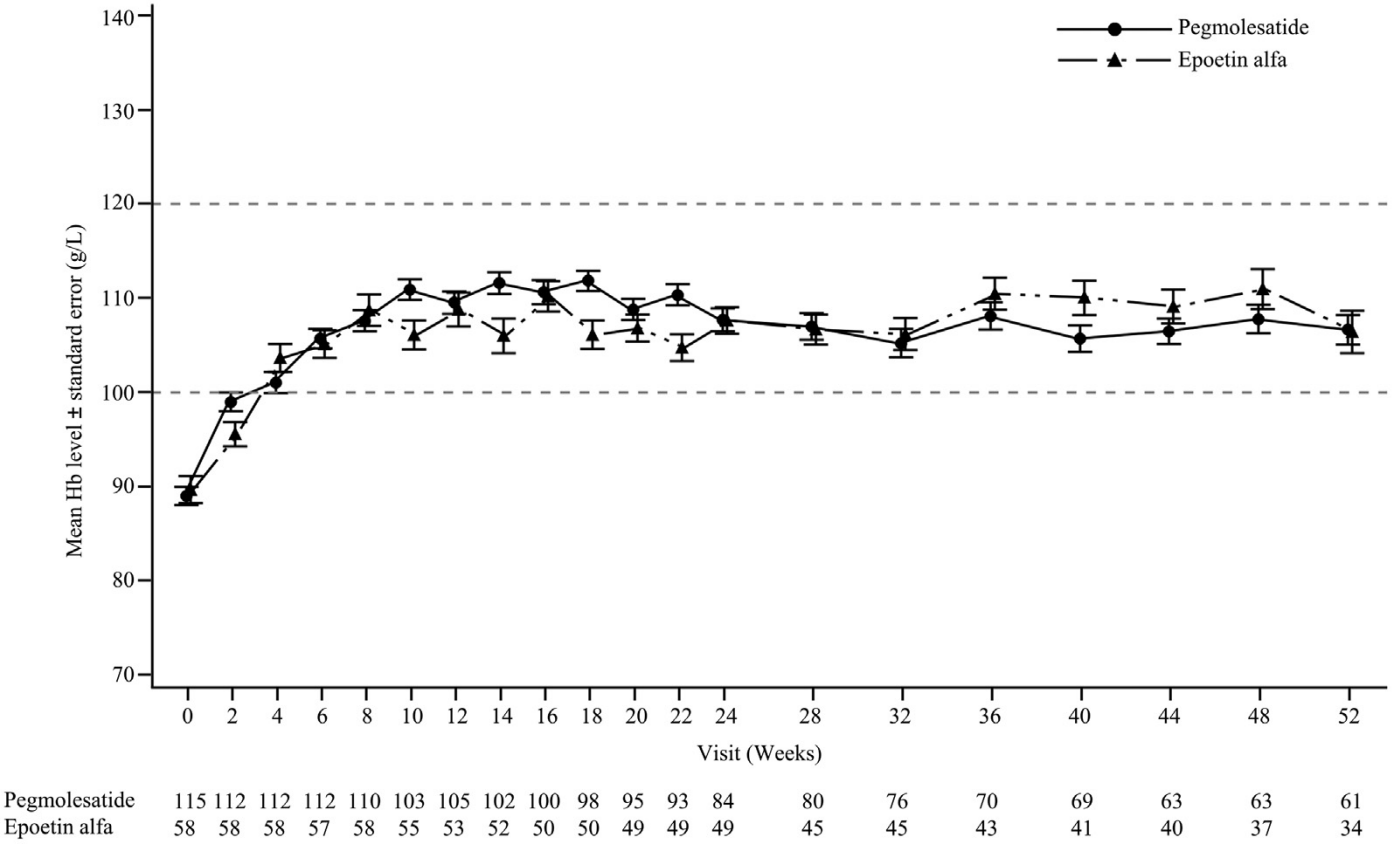
The mean Hb levels at each visit during the trial in the full analysis set. Hb, hemoglobin. SI conversion factors: To convert hemoglobin to g/l, multiply by 10.

**Figure 4:**
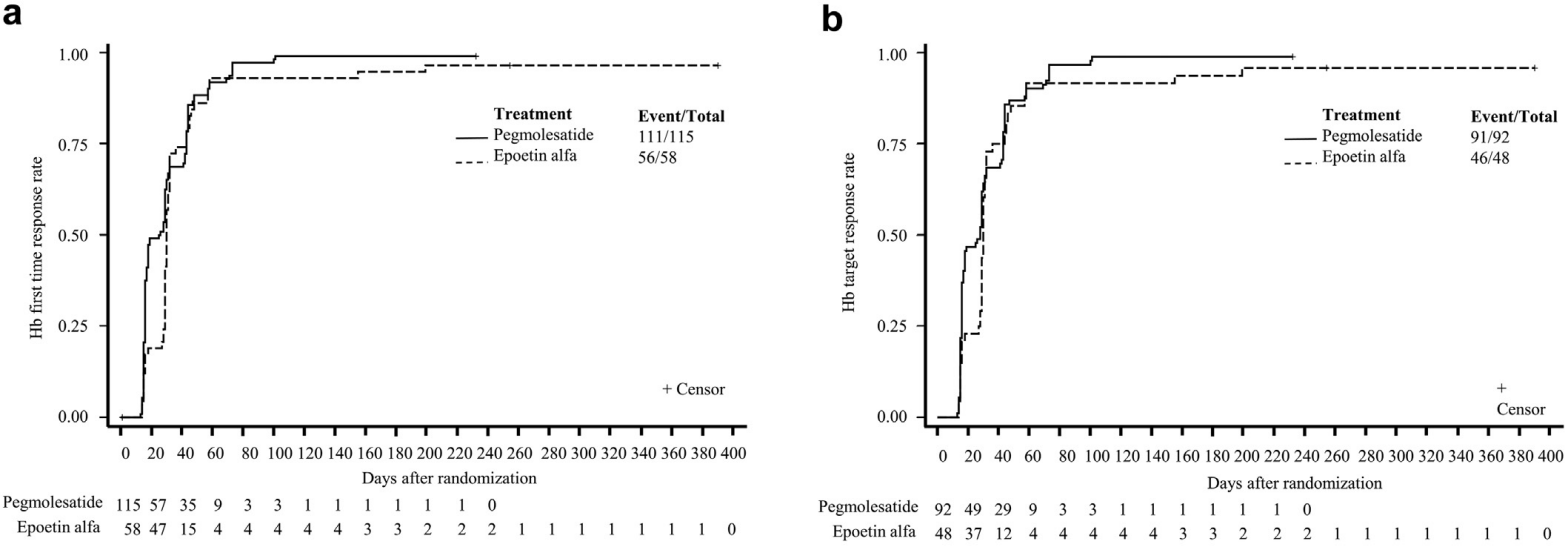
Time to achieve the first Hb response (a) and time to achieve the first Hb response target (b). Hb, hemoglobin.

The results of mean Hb levels at each visit throughout the trial showed that both groups exhibited an initial increase in Hb values at week 2 and the Hb values tend to be stable from week 8 to 52 (Figure 3). The pegmolesatide group demonstrated a median time to first Hb response of 25.5 (IQR: 16.0–43.0) days, with a response rate of 96.5%, whereas the epoetin alfa group had a median time of 30.0 (IQR: 29.0–43.0) days, with a response rate of 96.6%. The median time to achieve the first Hb response target was 43.0 (IQR: 17.0–47.5) days for the pegmolesatide group and 32.0 (IQR: 29.0–51.0) days for the epoetin alfa group, with response rates of 93.0% and 87.9%, respectively (Table 3 and Figure 4).

**Table 3:** Analysis of secondary efficacy end points (FAS)

The time to achieve the first Hb response and the time to achieve the first Hb response target^a^			
	Pegmolesatide (*n* = 115)	Epoetin alfa (*n* = 58)	*P* value
The time to achieve the first Hb response (d)			
Number of events (%)	111 (96.5)	56 (96.6)	0.10
Median (95% CI)	25.5 (17.0–29.0)	30.0 (29.0–32.0)	
P25 (95% CI)	16.0 (15.0–16.0)	29.0 (16.0–29.0)	
P75 (95% CI)	43.0 (31.0–44.0)	43.0 (32.0–48.0)	
HR (95% CI)^b^	1.26 (0.91–1.74)		
The time to achieve the first Hb response target (days)			
Number of events	107 (93.0)	51 (87.9)	0.30
Median (95% CI)	43.0 (29.0–43.0)	32.0 (30.0–43.0)	
P25 (95% CI)	17.0 (16.0–26.0)	29.0 (28.0–30.0)	
P75 (95% CI)	47.5 (44.0–69.0)	51.0 (43.0–155.0)	
HR (95% CI)^b^	1.23 (0.88–1.73)		

The actual mean dose of patients whose Hb maintained 100 g/l ≤ Hb ≤ 120 g/l during the efficacy evaluation period^c^			

	Pegmolesatide (mg/kg)	Epoetin alfa (IU/wk)	

Actual mean dose^d^			
*N*	67	42	
Mean (SD)	0.04 (0.02)	4789.8 (2205.5)	
M (Q1–Q3)	0.04 (0.03–0.05)	4383.0 (3500.0–6176.5)	
Min.–Max.	0–0.08	1500.0–9545.5	

CI, confidence interval; FAS, full analysis set; Hb, hemoglobin; HR, hazard ratio; Max., maximum; Min., minimum.

a The time to achieve the first Hb response, defined as an increase in Hb of ≥ 10 g/l from baseline at any time point during the trial; the time to achieve the first Hb response target, defined as follows: for patients with a baseline Hb ≥ 80 g/l, an increase in Hb of ≥ 10 g/l from baseline and reaching a Hb level of ≥ 100 g/l at any time during the trial; for patients with a baseline Hb < 80 g/l, an increase in Hb of ≥ 20 g/l from baseline at any time during the trial.

b Between-group comparisons were performed using log-rank tests; the Cox proportional risk model was used to estimate the between-group HR and its 95% CI, and baseline Hb was considered as a covariate in the model.

c Only subjects whose Hb was maintained between 100 g/l and 120 g/l during the efficacy evaluation period were analyzed for the actual mean dose.

d The actual mean dose of pegmolesatide was calculated in four weeks, and the mean dose of epoetin alfa was calculated in 1 week.

The percentage of participants receiving pegmolesatide and epoetin alfa maintaining their Hb level within the target range of 100 to 120 g/l during the efficacy evaluation period was statistically comparable (*P* = 0.09), with the actual median dose of 0.04 (IQR: 0.03–0.05) mg/kg per 4 wks and 4383.0 (IQR: 3500.0–6176.5) IU/wk (Table 3), respectively.

Following the initial 4 weeks of anemia correction, the pegmolesatide group consistently maintained a stable proportion of patients within the target Hb range, ranging from 60.7% to 70.0% from weeks 5 to 52. The maintenance ratio of the epoetin alfa group was similar to that of the pegmolesatide group, fluctuating between 63.8% and 73.5%, except for weeks 17 to 20, 21 to 24, and 45 to 48 (Supplementary Figure S2). In addition, during the initial 24 weeks, the proportion of participants with Hb levels exceeding 120 g/l was similar between groups (pegmolesatide vs. epoetin alfa: 42.6% vs. 39.7%); whereas during the extended treatment period, it was lower in pegmolesatide group (18.3% vs. 39.7%) (Supplementary Table S4). More participants receiving pegmolesatide maintained their Hb level within the range of 100 to 120 g/l after achieving the response target (pegmolesatide vs. epoetin alfa: 15.9%, 17 cases vs. 5.9%, 3 cases) (Supplementary Table S5).

### Safety Analysis

The overall safety profiles were similar between groups, with the incidence of AEs being 92.2% in the pegmolesatide group (106 cases) and 98.3% in the epoetin alfa group (57 cases) (Table 4 and Supplementary Table S6). AEs that occurred in at least 5% of participants in either group are listed in Supplementary Table S7. CKD progression was most reported in both groups (pegmolesatide vs. epoetin alfa: 30.4% vs. 31.0%). Hypertension was more frequent in the pegmolesatide group (27.8% vs. 20.7%), whereas hyperkalemia (25.2% vs. 34.5%), hyperphosphatemia (14.8% vs. 22.4%), peripheral oedema (6.1% vs. 24.1%), infectious pneumonia (2.6% vs. 13.8%), and cough (1.7% vs. 10.3%) occurred more frequently in the epoetin alfa group.

**Table 4:** Summary of AEs reported during the study (SS)

AEs	Pegmolesatide (*n* = 115)	Epoetin alfa (*n* = 58)
	No. of patients (%)	No. of patients (%)
Any AEs	106 (92.2)	57 (98.3)
Any treatment related AEs^a^	22 (19.1)	17 (29.3)
Treatment related AEs occurring in ≥ 2% of patients in either group		
Hypertension^b^	6 (5.2)	8 (13.8)
Prolonged QT interval on ECG	3 (2.6)	1 (1.7)
Hyperkalemia	2 (1.7)	4 (6.9)
Elevated aspartate aminotransferase	1 (0.9)	2 (3.4)
Pruritus	1 (0.9)	2 (3.4)
Any SAEs	44 (38.3)	27 (46.6)
SAEs occurring in ≥ 2% of patients in either group		
Chronic kidney disease^c^	29 (25.2)	18 (31.0)
Infectious pneumonia	1 (0.9)	3 (5.2)
Hyperkalemia	1 (0.9)	2 (3.4)
Acute heart failure	0	2 (3.4)
Composite safety events		
All-cause mortality	1 (0.9)	2 (3.4)
Stroke	0	0
Myocardial infarction	0	0
Other cardiovascular events		
Heart failure requiring hospitalization	0	3 (5.2)
Unstable angina requiring hospitalization	0	0

AEs, adverse events; ECG, electrocardiography; SAEs, serious adverse events; SS, safety set.

a Related to the study drug was defined as definitely related, possibly related, and undeterminable.

b Hypertension including preferred terms of “elevated blood pressure,” “high blood pressure,” and “poor blood pressure control.”

c Preferred terms of “chronic kidney disease” refers to the progression of chronic kidney disease.

Treatment-related AEs occurred in 22 patients in the pegmolesatide group and 17 in the epoetin alfa group (19.1% vs. 29.3%) (Table 4 and Supplementary Table S6). Among them, hypertension (5.2% vs. 13.8%), prolonged electrocardiographic QT intervals (2.6% vs. 1.7 %), hyperkalemia (1.7% vs. 6.9 %), elevated aspartate transaminase (0.9% vs. 3.4%), and pruritus (0.9% vs. 3.4%) were reported in over 2% of participants in any group. Most drug-related AEs were of grade 1 to 2 severity and resolved with or without intervention.

SAEs occurred in 44 patients in the pegmolesatide group and 27 patients in the epoetin alfa group (38.3% vs. 46.6%) (Table 4 and Supplementary Table S6). The SAE with the highest incidence in both groups was CKD progression (25.2% vs. 31.0%). Three deaths (0.9% vs. 3.4%) occurred during the study; they were unrelated to the study drugs (Supplementary Table S8).

The composite safety events that occurred consisted of all-cause deaths and were reported in 0.9% (1 case) in the pegmolesatide group and 3.4% (2 cases) in the epoetin alfa group (risk ratio = 0.26; 95% CI: 0.02–3.03) (Table 4). Three cases receiving epoetin alfa (5.2%) experienced other cardiovascular events.

During the study, 4 patients (3.5%) receiving pegmolesatide developed antidrug antibodies. Two (1.7%) of them had transient positivity at a single visit, and 2 experienced decreased efficacies alongside antibody production. Only 1 patient had neutralizing antibodies. No hypersensitivity reactions or significant safety risks were observed.

## Discussion

This multicenter, randomized, open-label, positive-controlled phase 3 study evaluated the efficacy and safety of pegmolesatide in ESA-naïve patients with NDD-CKD over 1 year. The participants had baseline characteristics that are comparable with other NDD-CKD studies.^18^ Pegmolesatide demonstrated noninferiority to epoetin alfa, meeting the primary efficacy end point. The findings were consistent across additional sensitivity analyses and prespecified subgroup analyses. Similar Hb improvements with pegmolesatide were observed among all patient subgroups, indicating the robustness of the conclusion.

Previous pivotal studies in patients with NDD-CKD have identified the attainment of the Hb response, in terms of both rate and time, as a key efficacy indicator for anemia-correction therapies.^19^^,^^20^ In the phase 3 trials for continuous erythropoietin receptor activator, it was observed that whereas the response rate was comparable with other ESAs, the median time to response was considerably more prolonged at 43 days.^19^^,^^21^ In our study, pegmolesatide showed a similar proportion for the response rate (96.5%) and a slightly shorter median time to response (25.5 days) compared with continuous erythropoietin receptor activator. The mean Hb level in the pegmolesatide group remained within the target range from the fourth week of treatment, thus meeting the objectives of initial ESA therapy and clinical practice requirements.^22^^,^^23^

In the present study, the target range for Hb levels was set at 100 to 120 g/l, following the 2017 National Institute for Health and Care Excellence guideline.^24^ Throughout the 52-week study period, both groups had a comparable proportion of patients with Hb levels within the target range. However, more patients receiving pegmolesatide maintained Hb levels within the 100 to 120 g/l range after anemia correction (pegmolesatide vs. epoetin alfa: 15.9% vs. 5.9%). This suggests that pegmolesatide demonstrated promising therapeutic efficacy in line with the current guidelines and may offer potential advantages in long-term stability.

The administered dosage of the investigational drugs in this study was adjusted based on Hb levels. Pegmolesatide consistently maintained an average dosage similar to the initial dosage throughout the study, indicating the rationality and stability of the administered dosage. Compared with epoetin alfa, the pegmolesatide group required fewer dose adjustments to achieve the target Hb level. Specifically, a higher proportion of patients in the pegmolesatide group did not require any dose adjustments and a lower proportion required multiple (≥ 5 times) dose adjustments (pegmolesatide vs. epoetin alfa: 13.9% vs. 25.5%). These findings suggest that the long-acting formulation of pegmolesatide can achieve an effective anemia correction and desirable Hb target stabilization with fewer dose adjustments, which may reduce the treatment gap for NDD renal anemia.

The safety profiles of pegmolesatide and epoetin alfa were highly similar in this study. No unexpected SAEs, hypersensitivity responses, or fatal events related to the study drugs were reported. The most common AEs and SAEs observed were CKD progression. The incidence of CKD progression was similar between groups, aligning with the baseline kidney function levels. Hypertension is a common AE associated with ESAs. In this study, it was slightly more commonly reported but less related to the study drug in the pegmolesatide group, given the higher baseline prevalence of hypertension in the pegmolesatide group and more patients experiencing blood pressure fluctuations being attributed to their underlying condition. The overall incidence of hypertension in this study was comparable to that reported in similar clinical trials of other long-acting ESAs.^17^^,^^25^

Given that previous studies have raised concerns about the increased risk of adverse cardiovascular outcomes associated with ESAs use in patients with CKD,^26^, ^27^, ^28^ cardiovascular risks were of particular interest in this study. A similar drug, peginesatide, demonstrated a higher incidence of composite safety end point events and sudden death in patients with NDD-CKD even though it showed to be equal to the comparator ESAs in patients with dialysis-dependent CKD.^17^^,^^29^ Some studies also suggested that a drug interaction with phenol-based preservatives and subvisible particles in the multidose vials formulation, could be causally linked to the serious adverse reactions (e.g., anaphylaxis) seen with peginesatide.^30^^,^^31^ However, in this study, no significant difference in adjudicated composite safety events and other cardiovascular events was found between the pegmolesatide and epoetin alfa groups. This indicates that pegmolesatide may not increase the risk of cardiovascular events in patients with CKD who are not on dialysis. In addition, pegmolesatide is marketed as preservative-free single-dose vials with simplified excipient formulation to mitigate risks of severe anaphylaxis. Further assessment of the cardiovascular safety and serious adverse reactions of pegmolesatide will be conducted in postmarketing studies.

The immunogenicity of pegmolesatide was within the expected range and comparable to other ESAs,^17^ with a 3.5% rate of antibody positivity and only 1 patient showing the presence of neutralizing antibodies. The presence of antidrug antibodies resulted in reduced efficacy in 2 patients (1.7%). No hypersensitivity reactions were reported. Thus, the immunogenicity of pegmolesatide is unlikely to significantly affect its safety and efficacy.

Certain limitations should be addressed in this study. First, the sample size and duration of follow-up may not be sufficient to draw definitive conclusions about long-term outcomes. Given that pegmolesatide has been approved in China only for 1 year, we currently lack long-term data on its safety and efficacy. A 3-year postmarketing surveillance study was conducted in China to fully evaluate the long-term efficacy, risks of cardiovascular and other AEs of pegmolesatide. Second, the open-label design could introduce potential biases from both patients and physicians. However, efforts were made to mitigate this concern through the involvement of an Independent Data Monitoring Committee that conducted safety data reviews in a blinded manner. Lastly, this multicenter study was conducted exclusively in the Chinese population; therefore, the generalizability of the findings to other ethnic groups is uncertain.

In conclusion, pegmolesatide demonstrated comparable therapeutic efficacy and safety profile to epoetin alfa in treating anemia in Chinese patients with NDD-CKD. The monthly subcutaneous administration of pegmolesatide proved noninferiority to the conventional weekly or biweekly regimen of epoetin alfa without increasing cardiovascular concern.

## Disclosure

The study was sponsored by Hansoh Pharmaceutical Group Co, Ltd (Shanghai, China). The sponsor collaborated with authors during study design, data analysis, data interpretation, report writing, and reviewed the manuscript before submission. XY, JX, AY, HQ, XP, WL, X-yH, QC, AZ, ST, and QW received a sponsorship from Hansoh Pharmaceutical Group Co, Ltd. CL, LH, XJ, AM, and FW are full-time employees of Hansoh Pharmaceutical Group Co, Ltd.

## Supplementary Material

Supplementary File (PDF) **Supplementary Materials and Methods. Data S1.** The details of 38 centers participated in this study in China. **Data S2.** List of inclusion and exclusion criteria. **Figure S1**. Prespecified subgroup analyses (FAS). **Figure S2.** The proportion of participants whose Hb levels maintained within the range of 100 to 120 g/l throughout the trial (FAS). **Table S1.** Drug exposure during the trial in both groups (SS). **Table S2.** Drug exposure in each period during the trial in both groups (SS). **Table S3.** Number of dose adjustment in both groups (SS). **Table S4.** Summary of Hb levels exceeding 120 g/l (FAS). **Table S5.** The proportion of Hb maintained 100 g/l ≤ Hb ≤ 120 g/l after achieving the Hb target (FAS). **Table S6.** Overview of overall adverse events (SS). **Table S7.** Occurrence of adverse events with an incidence of ≥ 5% (SS). **Table S8.** Serious adverse events leading to death (SS). **CONSORT Checklist.**

## Footnotes

Supplementary File (PDF)

**Supplementary Materials and Methods.**
**Data S1**. The details of 38 centers participated in this study in China. **Data S2**. List of inclusion and exclusion criteria. **Figure S1**. Prespecified subgroup analyses (FAS). **Figure S2.** The proportion of participants whose Hb levels maintained within the range of 100 to 120 g/l throughout the trial (FAS). **Table S1.** Drug exposure during the trial in both groups (SS). **Table S2.** Drug exposure in each period during the trial in both groups (SS). **Table S3.** Number of dose adjustment in both groups (SS). **Table S4.** Summary of Hb levels exceeding 120 g/l (FAS). **Table S5.** The proportion of Hb maintained 100 g/l ≤ Hb ≤ 120 g/l after achieving the Hb target (FAS). **Table S6.** Overview of overall adverse events (SS). **Table S7.** Occurrence of adverse events with an incidence of ≥ 5% (SS). **Table S8.** Serious adverse events leading to death (SS). **CONSORT Checklist**.
