# Supplementary material for: Randomized Trial of Pegmolesatide for the Treatment of Anemia in Patients With Nondialysis CKD
Source: Kidney Int Rep. 2024 Dec 6;10(3):720–9. doi: 10.1016/j.ekir.2024.12.002 (PMC11993201; doi:10.1016/j.ekir.2024.12.002)
Supplement: Supplementary File (PDF) — Supplementary Materials and Methods. Data S1. The details of 38 centers participated in this study in China. Data S2. List of inclusion and exclusion criteria. Figure S1. Prespecified subgroup analyses (FAS). Figure S2. The proportion of participants whose Hb levels maintained within the range of 100 to 120 g/l throughout the trial (FAS). Table S1. Drug exposure during the trial in both groups (SS). Table S2. Drug exposure in each period during the trial in both groups (SS). Table S3. Number of dose adjustment in both groups (SS). Table S4. Summary of Hb levels exceeding 120 g/l (FAS). Table S5. The proportion of Hb maintained 100 g/l ≤ Hb ≤ 120 g/l after achieving the Hb target (FAS). Table S6. Overview of overall adverse events (SS). Table S7. Occurrence of adverse events with an incidence of ≥ 5% (SS). Table S8. Serious adverse events leading to death (SS). CONSORT Checklist. [file mmc1.pdf]

Supplementary Material

Supplementary Materials and Methods

Immunogenicity analysis: The assessments of pegmolesatide anti-drug antibodies were performed before each dose, at week two and week four after dosing, and subsequently every four weeks until the completion of the study. Immunogenicity was accessed by a fully validated multi-tiered method to detect anti-drug antibody (ADA). This method was developed and validated at labcorp (shanghai, china) based on ELISA format, according to current regulatory guidance (FDA 2019, EMA 2017). Anti EPO-PEG rabbit IgG was used as positive control in this method, all samples were analyzed at a minimum required dilution (MRD) of 1:10. The assay sensitivity is 28.0 ng/mL for screening assay and 52.6 ng/mL for confirmatory assay. Drug tolerance results show that 70ng/mL of ADA can tolerate at least 200 ng/mL of pegmolesatide. Inter- and intra-assay precisions were all below 20% coefficient of variation (C.V) for screening and confirmatory assay tested for low, mid and high positive controls. The decrease in efficacy associated with antibody production was defined as a significant reduction in Hb levels while maintaining the same administered dose or requiring a substantial dose increase to sustain Hb levels.

Supplemental data 1. The details of 38 centres participated in this study in China

No.	Primary Investigator (PI)	Center	Enrolled patients
1	Xueqing Yu	Guangdong Provincial People's Hospital	12
2	Xiao Yang	The First Affiliated Hospital, Sun Yat-sen University	4
3	Lu Lv	The First Affiliated Hospital/ The First Clinical Medicine School of Guangdong Pharmaceutical University	3
4	Jianxin Wan	The First Affiliated Hospital of Fujian Medical University	1
5	Tiekun Yan	Tianjin Medical University General Hospital	1
6	Shaomei Li	The Second of Hebei Medical University	1
7	Zhonggao Xu	The First Hospital of Jilin University	0
8	Ping Luo	The Second Hospital of Jilin University	0
9	Hongguang Zheng	General Hospital of Northern Theater Command	6
10	Xiaoling Wang	The Second Hospital & Clinical Medical School, Lanzhou University	1
11	Zhanzheng Zhao	The First Affiliated Hospital of Zhengzhou University	5
12	Jing Li	The First Affiliated Hospital of Xinjiang Medical University	1
13	Menghua Chen	General Hospital of Ningxia Medical University	3
14	Wanhong Lu	The First Affiliated Hospital of Xi'an Jiangtong University	8
15	Yan La	Qingdao Municipal Hospital (Group)	1
16	Qin Wang	Shanghai Fengxian District Central Hospital	7
17	Hongyu Qiu	West China School of Medicine, West China Hospital of Sichuan University	13
18	Jianghua Chen	The First Affiliated Hospital Zhejiang University School of Medicine	0
19	Xiangyang Huang	Liuzhou Worker's Hospital	8
20	Xiaomei Peng	The People's Hospital of Guangxi Zhuang Autonomous Region	10
21	Qinkai Chen	The First Affiliated Hospital of Nanchang University	8
22	Li Wang	Sichuan Provincial People's Hospital	4
23	Aimin Zhong	Jiangxi Provincial People's Hospital	8
24	Xiaoxia Wang	TongRen Hospital, Shanghai Jiaotong University School of Medicine	3
25	Rong Li	The Second Hospital of Tianjin Medical University	6
26	Yueqin Ren	Linyi People's Hospital	6
27	Zunsong Wang	Shandong Provincial Qianfoshan Hospital, The First Affiliated Hospital of Shandong First Medical University	1
28	Deguang Wang	The Second Hospital of Anhui Medical University	4
29	Guoyuan Lu	The First Affiliated Hospital of Soochow University	6
30	Hongli Lin	The First Affiliated Hospital of Dalian Medical University	1
31	Shuifu Tang	The First Affiliated Hospital of Guangzhou University of Chinese Medicine	8
32	Chengyun Xu	The Second Affiliated Hospital of Nanchang University	6
33	Shuguang Qin	Guangzhou First People's Hospital	3

No.	Primary Investigator (PI)	Center	Enrolled patients
34	Deqiong Xie	The Second People's Hospital of Yibin	5
35	Minghao Guo	The First Affiliated Hospital of Xinxiang Medical University	0
36	Aicheng Yang	Wuyi Chinese Medicine Hospital of Jiangmen	15
37	Yuou Xia	Siping City Central People’s Hospital	5
38	Hua Zhou	Shengjing Hospital of China Medical University	1

Supplemental data 2. List of inclusion and exclusion criteria

Subjects will be enrolled if all of the following criteria are met:

1) Patients who are 18-70 years old (including cut points), regardless of gender;
2) Subjects who have chronic kidney disease stage of CKD3-5, have estimated glomerular filtration rate (eGFR) < 60 mL/min/1.73 m^2 during the screening period (Note: The eGFR is evaluated using the CKD-EPI formula), and have no dialysis treatment plan during the trial;
3) Subjects who have not received any ESA treatment within 12 weeks prior to randomization, and have the Hb measured value within the range of 60 g/L ≤ Hb < 100 g/L during the screening period;
4) Subjects who have transferrin saturation (TSAT) level ≥ 20% and serum ferritin (SF) ≥ 100 ng/mL, serum folate level ≥ lower limit of normal, and vitamin B12 ≥ lower limit of normal during the screening period;
5) Understand the study procedures and voluntarily sign the informed consent form (ICF) in writing.

Subjects are not selected in case of any of the following circumstances:

1) Pregnant or breastfeeding female patients or women of childbearing potential who test positive for the baseline pregnancy test (3 days prior to the first dose). Only if one of the following criteria to prove that there is no risk of pregnancy is met:
   a. Women who are postmenopausal (defined as women aged over 50, and having amenorrhea for at least 12 months after all hormone therapies being stopped);
   b. For women no older than 50 years old, if the amenorrhea has lasted at least 12 months after stopping all hormone therapies, and the luteinizing hormone (LH) and follicle stimulating hormone (FSH) levels are within the laboratory postmenopausal reference value range, the risk for pregnancy is not considered;
   c. Subjects who have received irreversible sterilization, including hysterectomy, bilateral oophorectomy or bilateral fallopian tube resection, except for bilateral fallopian tube ligation;
2) Subjects who have received red blood cell or whole blood transfusion therapy within 12 weeks prior to randomization (Note: All "transfusion therapy" described in this protocol refers specifically to transfusion therapy of red blood cell component or whole blood);
3) Subjects who are known to be intolerant to erythropoiesis-stimulating agents (ESAs), injected iron or polyethylene glycol molecules;
4) Subjects who are known to have the history of allergy, including but not limited to a history of definite drug or food allergy, or medically diagnosed anaphylactic disease (such as allergic asthma, allergic purpura, allergic rhinitis, allergic dermatitis, etc.);
5) Subjects who are known to have hematologic disorders (both congenital and acquired, such as thalassemia, Fanconi anemia, aplastic anemia, myelodysplastic syndrome, hemolytic anemia, and bleeding and coagulation disorders) or other causes of anemia other than CKD (such as gastrointestinal bleeding, hookworm disease, or celiac disease);
6) Subjects who are known to have autoimmune system disorders other than glomerulonephritis (such as rheumatoid arthritis, systemic lupus erythematosus, antineutrophil cytoplasmic antibody-associated vasculitis, diffuse toxic goiter (Graves' disease), or autoimmune hypothyroidism);
7) Subjects who had significant infection within 4 weeks prior to randomization as determined by the investigator;
8) Subjects who have chronic, uncontrollable or symptomatic inflammation at the discretion of the investigator;
9) Subjects who have uncontrollable or symptomatic secondary hyperparathyroidism at the discretion of the nvestigator, or who have blood iPTH > 600 pg/mL during the screening period;
10) Subjects who have poor control of blood pressure within 4 weeks prior to randomization at the discretion of the investigator (please refer to Appendix 5 for specific determination criteria);
11) Subjects who have cardiac function NYHA class III or IV assessed during screening;
12) Subjects who are known to have decompensated liver cirrhosis or have abnormality in any of the following tests within 6 months prior to randomization (ALT ≥ 2 × upper limit of normal, AST ≥ 2 × upper limit of normal, DBIL ≥ 2 × upper limit of normal);
13) Subject who are HIV positive;

14) Subjects who have clinically significant diseases or conditions (such as acute myocardial infarction, severe or unstable coronary artery disease, malignant arrhythmias, stroke, epilepsy, or other neuropsychiatric disorders) within 6 months prior to screening that, in the judgment of the investigator, may interfere with evaluations or follow-up visit;
15) Subjects who are receiving and require long-term immunosuppressive therapy;
16) Subjects who have the history of prior or current malignancy (excluding resected non-melanoma skin cancer and carcinoma in situ);
17) Subjects who have the expected survival of < 12 months;
18) Subjects who have the history of organ or hematopoietic stem cell transplantation (except renal transplant) prior to screening;
19) Subjects who plan to get elective surgery during the study;
20) Subjects who have the plan for pregnancy or donating sperm or eggs within 4 months after the end of the trial;
21) Subjects who participated in any drug clinical trial other than this trial and took investigational drugs within 12 weeks prior to inclusion, or have participated in other device clinical trials and received device treatment, or plan to receive any other drug trial or device trial during the trial;
22) Subjects who have other conditions that, in the judgment of the investigator, make the subject unsuitable for participation in the study.

Supplementary Table S1. Drug exposure during the trial in both groups (SS)

Indicators		Pegmolesatide (N = 115)	Epoetin alfa (N = 58)
Exposure time (weeks)	N (Nmiss)	115 (0)	58 (0)
	Mean (SD)	38.3 (16.2)	40.0 (14.8)
	M (Q1~Q3)	51.7 (23.9~52.0)	48.7 (30.6~50.7)
	Min~Max	4.0~53.3	4.4~51.9
Actual mean dose	N (Nmiss)	115 (0)	58 (0)
	Mean (SD)	2.5 (0.9)	5113.4 (2127.04)
	M (Q1~Q3)	2.5 (1.8~3.1)	4802.6 (3571.8~7000.0)
	Min~Max	0.7~5.3	1750.0~8936.2
Actual mean dose (mg/kg)	N (Nmiss)	115 (0)	-
	Mean (SD)	0.04 (0.01)	-
	M (Q1~Q3)	0.04 (0.03~0.05)	-
	Min~Max	0.01~0.08	-

Note: Duration of exposure (weeks) of pegmolesatide = (date of last dose - date of first dose after week 0 + 28) / 7; duration of exposure (weeks) of epoetin alfa = (date of last dose - date of first dose after week 0+3) / 7; actual mean dose = total dose administered/duration of exposure; in mg/ per-4-week for the pegmolesatide group and in unit/per week for the epoetin alfa group; actual mean dose (mg/kg) = total of each (dose/pre-dose body weight)/duration of exposure; units for pegmolesatide group are mg/kg/4w; mean pegmolesatide exposure was calculated in 4-week time units, and mean epoetin alfa exposure was calculated in 1-week time units.

Supplementary Table S2. Drug exposure in each period during the trial in both groups (SS)

Indicators		[a]Pegmolesatide (N = 115)	[b]Epoetin alfa (N = 58)
First dose	N (Nmiss)	115 (0)	58 (0)
	Mean (SD)	0.04 (0.001)	6000.0 (0.0)
	M (Q1~Q3)	0.04 (0.04~0.04)	6000.0 (6000.0~6000.0)
	Min~Max	0.04~0.05	6000.0~6000.0
Actual mean dose during dose-titration period	N (Nmiss)	115 (0)	58 (0)
	Mean (SD)	0.04 (0.01)	5392.3 (1812.0)
	M (Q1~Q3)	0.04 (0.03~0.05)	5511.7 (4017.4~6595.0)
	Min~Max	0.02~0.07	2413.8~8950.8
Actual mean dose during efficacy evaluation period	N (Nmiss)	89 (26)	50 (8)
	Mean ± SD	0.04 (0.02)	5509.8 (2796.7)
	M (Q1~Q3)	0.04 (0.03~0.05)	5052.9 (3559.3~7118.6)
	Min~Max	0.01~0.09	1500.0~14000.0
Actual mean dose during extended period	N (Nmiss)	75 (40)	45 (13)
	Mean ± SD	0.04 (0.02)	5412.8 (2956.8)
	M (Q1~Q3)	0.04 (0.03~0.06)	5011.4 (2942.7~8451.2)
	Min~Max	0.01~0.09	969.2~10500.0

Note: [a] Pegmolesatide is measured in mg/kg/per 4-week；[b]epoetin alfa is measured in unit/per week; duration of exposure of pegmolesatide in each period (weeks) = (date of last dose in each period - date of first dose in each period + 28) / 7; duration of exposure of epoetin alfa in each period (weeks) = (date of last dose in each period - date of first dose in each period +3) / 7; actual mean dose during each period = total dose administered in each period / duration of exposure in each period; in mg/per-4-week for the pegmolesatide group and in unit/per week for the epoetin alfa group; actual mean dose during each period (mg/kg) = total of each (dose/pre-dose body weight) in each period / duration of exposure in each period; units for pegmolesatide group are mg/kg/4w; mean pegmolesatide exposure was calculated in 4-week time units, and mean epoetin alfa exposure was calculated in 1-week time units.

Supplementary Table S3. Number of dose adjustment in both groups (SS)

Indicator		Pegmolesatide (N = 115)	Epoetin alfa (N = 58)
Dose adjustments, n (%)	0	21 (18.3)	2 (3.4)
	1	23 (20.0)	9 (15.5)
	2	22 (19.1)	14 (24.1)
	3	25 (21.7)	11 (19.0)
	4	8 (7.0)	7 (12.1)
	≥ 5	16 (13.9)	15 (25.9)
	Total	115 (100.0)	58 (100.0)
	Mean (SD)	2.4 (1.9)	3.1 (1.7)

Note: Multiple dose adjustments may occur in the same subject, counted as the maximum number of adjustments.

Supplementary Table S4. Summary of Hb levels exceeding 120 g/L (FAS)

	Pegmolesatide (N = 115)	Epoetin alfa (N = 58)
	**No. of patients (%)**	**No. of patients (%)**
Overall	53 (46.1)	33 (56.9)
The initial 24 weeks	49 (42.6)	23 (39.7)
The last 24 weeks	21 (18.3)	23 (39.7)

Note: Subjects with at least one visit exceeding 120 g/L were included in the analysis

Supplementary Table S5. The proportion of Hb maintained 100 g/L ≤ Hb ≤ 120 g/L after achieving the Hb target (FAS)

	**Pegmolesatide (n = 107)**	**Epoetin alfa (n = 51)**	***P* value**
No. of maintenance	17	3	0.05
Proportion of maintenance	15.9	5.9	
The rate difference	11.23		
95% CI	(1.88, 20.59)		

Note: Maintenance was defined as a Hb level of 100 to 120 g/L at all visits after the response target was met; rate differences and two-sided 95% CIs were derived from the CMH test with adjustment for baseline Hb group (≤ 89 g/L vs. ≥ 90 g/ L)

Supplementary Table S6. Overview of overall adverse events (SS)

	Pegmolesatide (N = 115)	Epoetin alfa (N = 58)
	**No. of patients (%)**	**No. of patients (%)**
All AEs	106 (92.2)	57 (98.3)
≥ grade 3 AEs	54 (47.0)	28 (48.3)
AEs related to the study drug	22 (19.1)	17 (29.3)
≥ grade 3 AEs and related to the study drug	3 (2.6)	6 (10.3)
AEs leading to dose reduction	2 (1.7)	1 (1.7)
AEs leading to dose reduction and related to the study drug	1 (0.9)	1 (1.7)
AEs leading to suspension of medication	3 (2.6)	4 (6.9)
AEs leading to suspension of medication and related to the study drug	1 (0.9)	1 (1.7)
AEs leading to permanent discontinuation of the drug	25 (21.7)	12 (20.7)
AEs leading to permanent discontinuation of the drug and related to the study drug	0	0
Serious AEs (SAEs)	44 (38.3)	27 (46.6)
SAEs related to the study drug	0	0
SAEs leading to death	1 (0.9)	2 (3.4)
SAEs leading to death and related to the study drug	0	0
SAEs leading to withdrawal from the trial	25 (21.7)	12 (20.7)
SAEs leading to withdrawal from the trial and related to the study drug	0	0

Note: Adverse events related to the study drug were defined as adverse events that were "definitely related/possibly related/could not be determined".

Supplementary Table S7. Occurrence of adverse events with an incidence of ≥ 5% (SS)

Preferred terms	Pegmolesatide (N = 115)	Epoetin alfa (N = 58)
	No. of patients (%)	No. of patients (%)
Chronic Kidney Disease [a]	35 (30.4)	18 (31.0)
Hypertension [b]	32 (27.8)	12 (20.7)
Hyperkalaemia	29 (25.2)	20 (34.5)
Upper Respiratory Tract Infections	29 (25.2)	14 (24.1)
Hyperphosphatemia	17 (14.8)	13 (22.4)
Hyperlipidaemia	15 (13.0)	6 (10.3)
Urinary tract infection	10 (8.7)	8(13.8)
Dizziness	10 (8.7)	5 (8.6)
Elevated creatinine	9 (7.8)	5 (8.6)
Pruritus	9 (7.8)	5 (8.6)
Nausea	8 (7.0)	6 (10.3)
Diarrhoea	8 (7.0)	3 (5.2)
Peripheral oedema	7 (6.1)	14 (24.1)
headache	7 (6.1)	4 (6.9)
insomnia	6 (5.2)	4 (6.9)
Prolonged QT interval on ECG	5 (4.3)	5 (8.6)
constipation	5 (4.3)	4 (6.9)
Metabolic acidosis	5 (4.3)	4 (6.9)
Hypocalcaemia	5 (4.3)	6 (10.3)
Hyperuricemia	5 (4.3)	6 (10.3)
Nasopharyngitis	4 (3.5)	4 (6.9)
Vomiting	4 (3.5)	3 (5.2)
Hypokalaemia	3 (2.6)	4 (6.9)
Loss of appetite	3 (2.6)	4 (6.9)
Infectious Pneumonia	3 (2.6)	8 (13.8)
Elevated AST	3 (2.6)	4 (6.9)
Nephrolithiasis	3 (2.6)	3 (5.2)
Kidney cysts	3 (2.6)	3 (5.2)
Abdominal Pain	3 (2.6)	3 (5.2)
Upper Abdominal Pain	3 (2.6)	3 (5.2)
Fever	3 (2.6)	4 (6.9)
Back Pain	3 (2.6)	5 (8.6)
Hypercalcemia	2 (1.7)	3 (5.2)
Elevated ALT	2 (1.7)	4 (6.9)
ST-segment abnormalities on ECG	2 (1.7)	3 (5.2)
Fatigue	2 (1.7)	3 (5.2)
Cough	2 (1.7)	6 (10.3)

Note: [a] Chronic kidney disease refers to the progression of chronic kidney disease; [b] hypertension includes elevated blood pressure, high blood pressure, and poor blood pressure control.

Supplementary Table S8. Serious adverse events leading to death (SS)

Serious adverse events	Pegmolesatide (N=115)	Epoetin alfa (N=58)
	**No. of patients (%)**	**No. of patients (%)**
All SAEs leading to death	1 (0.9)	2 (3.4)
Infectious and Invasive Diseases [a]	1 (0.9)	0
Diabetic foot infections	1 (0.9)	0
Systemic diseases and reactions at the site of administration [a]	0	1 (1.7)
Sudden death	0	1 (1.7)
Heart Organ Diseases [a]	0	1 (1.7)
Chronic heart failure	0	1 (1.7)

Note: [a] This is a system organ class (e.g. ‘Infectious and Invasive Diseases’ is the system organ class and ‘diabetic foot infections’ is the preferred term)

Supplementary Figure S1. Pre-specified subgroup analyses (FAS)

Subgroup	Pegmolesatide	Epoetin alfa	LS mean difference (95% CI) (Pegmolesatide - Epoetin alfa)
All	1.92(1.74,2.10)	1.54(1.29,1.79)	0.38(0.07,0.69)
Baseline Hb			
<=8.9 g/dL	2.36(2.07,2.65)	1.84(1.41,2.27)	0.52(0.00,1.04)
>=9.0 g/dL	1.49(1.28,1.70)	1.24(0.96,1.52)	0.25(-0.10,0.61)
NYHA Class			
0-I class	1.91(1.73,2.10)	1.62(1.37,1.87)	0.29(-0.02,0.61)
II class	1.82(1.41,2.22)	1.30(0.69,1.92)	0.51(-0.23,1.25)
Gender			
Male	1.87(1.57,2.18)	1.43(1.04,1.83)	0.44(-0.06,0.94)
Female	1.96(1.74,2.17)	1.64(1.31,1.97)	0.31(-0.08,0.71)

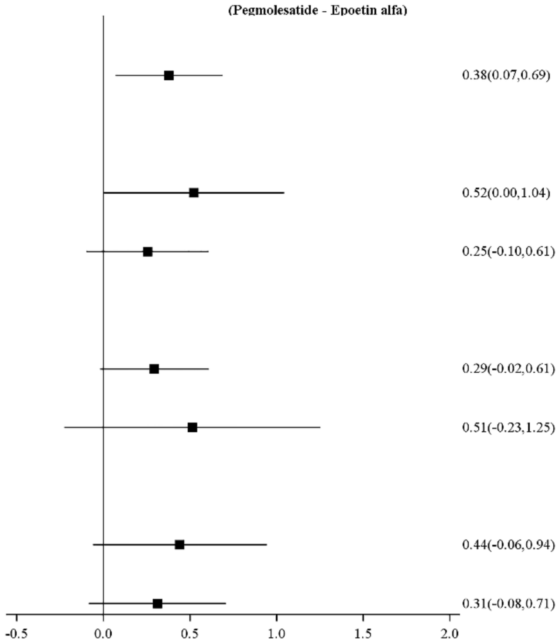

Supplementary Figure S2. The proportion of participants whose Hb levels maintained within the range of 100-120 g/L throughout the trial (FAS)

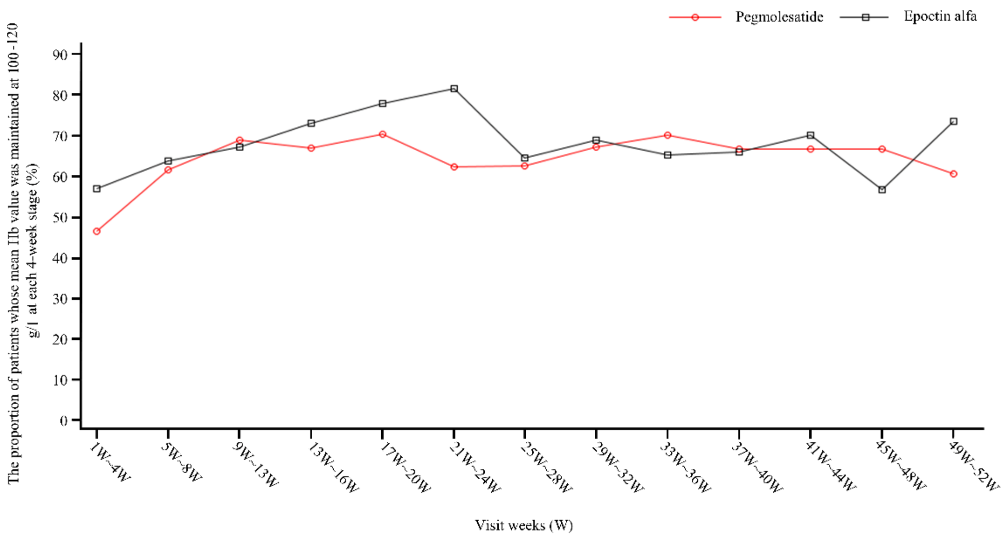

CONSORT 2010 checklist of information to include when reporting a randomised trial*

Section/Topic	Item No	Checklist item	Reported on page No
**Title and abstract**			
	1a	Identification as a randomised trial in the title	1
	1b	Structured summary of trial design, methods, results, and conclusions (for specific guidance see CONSORT for abstracts)	3
**Introduction**			
Background and objectives	2a	Scientific background and explanation of rationale	4
	2b	Specific objectives or hypotheses	4
**Methods**			
Trial design	3a	Description of trial design (such as parallel, factorial) including allocation ratio	5,6
	3b	Important changes to methods after trial commencement (such as eligibility criteria), with reasons	NA
Participants	4a	Eligibility criteria for participants	5
	4b	Settings and locations where the data were collected	5
Interventions	5	The interventions for each group with sufficient details to allow replication, including how and when they were actually administered	6
Outcomes	6a	Completely defined pre-specified primary and secondary outcome measures, including how and when they were assessed	6-7
	6b	Any changes to trial outcomes after the trial commenced, with reasons	NA
Sample size	7a	How sample size was determined	7
	7b	When applicable, explanation of any interim analyses and stopping guidelines	NA
Randomisation:			
Sequence generation	8a	Method used to generate the random allocation sequence	6
	8b	Type of randomisation; details of any restriction (such as blocking and block size)	6
Allocation concealment mechanism	9	Mechanism used to implement the random allocation sequence (such as sequentially numbered containers), describing any steps taken to conceal the sequence until interventions were assigned	6
Implementation	10	Who generated the random allocation sequence, who enrolled participants, and who assigned participants to interventions	6
Blinding	11a	If done, who was blinded after assignment to interventions (for example, participants, care providers, those	6

		assessing outcomes) and how	6
	11b	If relevant, description of the similarity of interventions	NA
Statistical methods	12a	Statistical methods used to compare groups for primary and secondary outcomes	9
	12b	Methods for additional analyses, such as subgroup analyses and adjusted analyses	8-9
**Results**			
Participant flow (a diagram is strongly recommended)	13a	For each group, the numbers of participants who were randomly assigned, received intended treatment, and were analysed for the primary outcome	9
	13b	For each group, losses and exclusions after randomisation, together with reasons	9
Recruitment	14a	Dates defining the periods of recruitment and follow-up	9
	14b	Why the trial ended or was stopped	NA
Baseline data	15	A table showing baseline demographic and clinical characteristics for each group	9
Numbers analysed	16	For each group, number of participants (denominator) included in each analysis and whether the analysis was by original assigned groups	9
Outcomes and estimation	17a	For each primary and secondary outcome, results for each group, and the estimated effect size and its precision (such as 95% confidence interval)	9-11
	17b	For binary outcomes, presentation of both absolute and relative effect sizes is recommended	NA
Ancillary analyses	18	Results of any other analyses performed, including subgroup analyses and adjusted analyses, distinguishing pre-specified from exploratory	9-11
Harms	19	All important harms or unintended effects in each group (for specific guidance see CONSORT for harms)	NA
**Discussion**			
Limitations	20	Trial limitations, addressing sources of potential bias, imprecision, and, if relevant, multiplicity of analyses	15
Generalisability	21	Generalisability (external validity, applicability) of the trial findings	15
Interpretation	22	Interpretation consistent with results, balancing benefits and harms, and considering other relevant evidence	13-15
**Other information**			
Registration	23	Registration number and name of trial registry	5
Protocol	24	Where the full trial protocol can be accessed, if available	Review file
Funding	25	Sources of funding and other support (such as supply of drugs), role of funders	16

Citation: Schulz KF, Altman DG, Moher D, for the CONSORT Group. CONSORT 2010 Statement: updated guidelines for reporting parallel group randomised trials. BMC Medicine. 2010;8:18. © 2010 Schulz et al. This is an Open Access article distributed under the terms of the Creative Commons Attribution License (http://creativecommons.org/licenses/by/2.0), which permits unrestricted use, distribution, and reproduction in any medium, provided the original work is properly cited.

*We strongly recommend reading this statement in conjunction with the CONSORT 2010 Explanation and Elaboration for important clarifications on all the items. If relevant, we also recommend reading CONSORT extensions for cluster randomised trials, non-inferiority and equivalence trials, non-pharmacological treatments, herbal interventions, and pragmatic trials. Additional extensions are forthcoming: for those and for up-to-date references relevant to this checklist, see www.consort-statement.org.
